# Photocontrol of Axillary Bud Outgrowth by MicroRNAs: Current State-of-the-Art and Novel Perspectives Gained From the Rosebush Model

**DOI:** 10.3389/fpls.2021.770363

**Published:** 2022-01-31

**Authors:** Julie Mallet, Patrick Laufs, Nathalie Leduc, José Le Gourrierec

**Affiliations:** ^1^University of Angers, Institut Agro, INRAE, IRHS, SFR QUASAV, Angers, France; ^2^Université Paris-Saclay, INRAE, AgroParisTech, Institut Jean-Pierre Bourgin (IJPB), Versailles, France

**Keywords:** branching, light control, lateral meristem, post-transcriptional regulation, small RNAs, rose

## Abstract

Shoot branching is highly dependent on environmental factors. While many species show some light dependence for branching, the rosebush shows a strict requirement for light to allow branching, making this species an excellent model to further understand how light impinges on branching. Here, in the first part, we provide a review of the current understanding of how light may modulate the complex regulatory network of endogenous factors like hormones (SL, IAA, CK, GA, and ABA), nutrients (sugar and nitrogen), and ROS to control branching. We review the regulatory contribution of microRNAs (miRNAs) to branching in different species, highlighting the action of such evolutionarily conserved factors. We underline some possible pathways by which light may modulate miRNA-dependent regulation of branching. In the second part, we exploit the strict light dependence of rosebush for branching to identify putative miRNAs that could contribute to the photocontrol of branching. For this, we first performed a profiling of the miRNAs expressed in early light-induced rosebush buds and next tested whether they were predicted to target recognized regulators of branching. Thus, we identified seven miRNAs (miR156, miR159, miR164, miR166, miR399, miR477, and miR8175) that could target nine genes (*CKX1/6*, *EXPA3*, *MAX4*, *CYCD3;1*, *SUSY*, *6PFK*, *APX1*, and *RBOHB1*). Because these genes are affecting branching through different hormonal or metabolic pathways and because expression of some of these genes is photoregulated, our bioinformatic analysis suggests that miRNAs may trigger a rearrangement of the regulatory network to modulate branching in response to light environment.

## Introduction

Important agronomic traits such as yield, visual and sanitary qualities, harvest index and even organoleptic quality are determined by plant architecture in general and shoot branching in particular (Boumaza et al., 2010; Garbez et al., 2015; Zhu and Wagner, 2020). A lot of research efforts are therefore produced to decipher mechanisms that control branching (Rameau et al., 2015; Roman et al., 2016; Le Moigne et al., 2018; Schneider et al., 2019; Wang M. et al., 2020; Barbier et al., 2021).

Branching relies on the ability of an axillary bud, a structure containing a miniature shoot comprising a meristem, short internodes and immature leaves, to break dormancy and to grow through cell proliferation and expansion into a new branch. Dormancy is complex and includes endo-, para-, and eco-dormancies that can in part overlap (Lang et al., 1987; Cline, 1997). While endodormancy is controlled by mechanisms endogenous to the bud, paradormancy is due to the control of other organs on a given bud, as it is the case in apical dominance. Apical dominance can be lifted by stem beheading (Dun et al., 2006) or by exogenous application of chemical products (Ophir et al., 2009; Suttle, 2009; Walton et al., 2009). Ecodormancy relies on environmental control over a bud. Bud ability to grow out is therefore controlled both by internal factors [i.e., genetic background, hormones, metabolites, reactive oxygen species (ROS)] (Girault et al., 2010; Mason et al., 2014; Barbier et al., 2015; Li-Marchetti et al., 2015; Porcher et al., 2021) and also external ones. Among them, nutrients and water availability (Demotes-Mainard et al., 2013), temperature (Djennane et al., 2014), and light (Djennane et al., 2014; Roman et al., 2016; Porcher et al., 2021) are major determinants. Responses to abiotic factors are indeed critical for the plant to adapt its own development to resource availability, seasons and environmental stresses and to compete with other plants.

Light signal is particularly informative for the plant since it varies in intensity, quality, direction, and duration (Leduc et al., 2014). The shade avoidance syndrome triggered by decreased photosynthetic radiations and R/FR (Red/Far Red) ratio leads for example to axillary bud outgrowth inhibition. Dependence on light for axillary bud growth is varying from one species to another. Some species are able to show axillary bud growth in darkness where in the rosebush, light is particularly essential for this. Indeed, in this species axillary bud outgrowth and organogenesis are totally inhibited and no axillary branches are produced in the absence of light (Girault et al., 2008). Beside rosebush’s primary importance among ornamentals, this response has also made it an excellent model for exploring light control of axillary bud outgrowth in plants (Demotes-Mainard et al., 2016; Huché-Thélier et al., 2016).

At the molecular level, most knowledge on axillary bud outgrowth and Its photocontrol has been gained at the transcriptional level in the rosebush (Girault et al., 2010; Djennane et al., 2014; Roman et al., 2016; Schneider et al., 2019; Porcher et al., 2020, 2021). Yet, as for other developmental processes, post-transcriptional regulations are likely important and need to be further explored. Some studies have revealed post-transcriptional regulation in axillary bud outgrowth control with post-transcriptional regulation of Rosa *BRANCHED1* (*RhBRC1*) in interaction with sugars and regulation of its 3′UTR region with the potential role of *PUMILIO RNA-BINDING PROTEIN FAMILY 4* (*RhPUF4*) (Wang et al., 2019b).

MicroRNA (miRNA) regulation is a major component of post-transcriptional regulation of all biological processes in eukaryotes. First discovered in *Caenorhabditis elegans* (Wightman et al., 1993), miRNAs are small (20–21 nucleotides) single stranded non-coding RNAs. In *Arabidopsis thaliana*, 728 mature miRNAs have been identified (Kozomara et al., 2019). MiRNAs modulate the expression of their target genes through binding to their mRNA, causing either transcript cleavage or translation inhibition (Voinnet, 2009). Some miRNAs and their targets show a high conservation within plants while non-conserved ones can be found only in a group of plants, a species or even be specific to a landrace (Rajagopalan et al., 2006; Fahlgren et al., 2007). Conserved miRNAs target preferentially genes coding for transcription factors that play important roles in developmental control, while non-conserved target genes code for much more diverse functions (Zhang and Zhang, 2012).

Some knowledge has been gained on the roles of miRNAs in meristem initiation (see reviews by Zhang et al., 2006; Wang et al., 2011; Wu, 2013; Li and Zhang, 2016; Liu et al., 2018) as well as on leaf organogenesis (Pulido and Laufs, 2010; Maugarny-Calès and Laufs, 2018; Yang et al., 2018), two processes that contribute to axillary bud initiation and development (Sussex and Kerk, 2001). However, little is known about the roles of miRNAs during axillary bud outgrowth *per se*. Here, we provide a survey of the current knowledge on this subject, first sketching how the main mechanisms regulating axillary bud outgrowth may be connected to light control. Next, we provide a comprehensive view of axillary bud outgrowth regulation by miRNAs and, exploring the wider literature, we provide hypotheses on how light controls miRNAs activity and may contribute to the photocontrol of axillary bud outgrowth. In a second part, based on our expertise in rosebush and using *in silico* analysis based on the miRNA profiling we performed, we further explored the rose genome to identify and discuss novel miRNAs-gene target couples that may play a potential role in the regulation of axillary bud outgrowth and in its photocontrol in rosebush. Thus, we provide novel hypotheses on the miRNA-mediated regulation of bud growth photocontrol on which future research may stand.

## Axillary Bud Outgrowth and its Light Control: Current Knowledge on Main Actors

**FIGURE 1:**
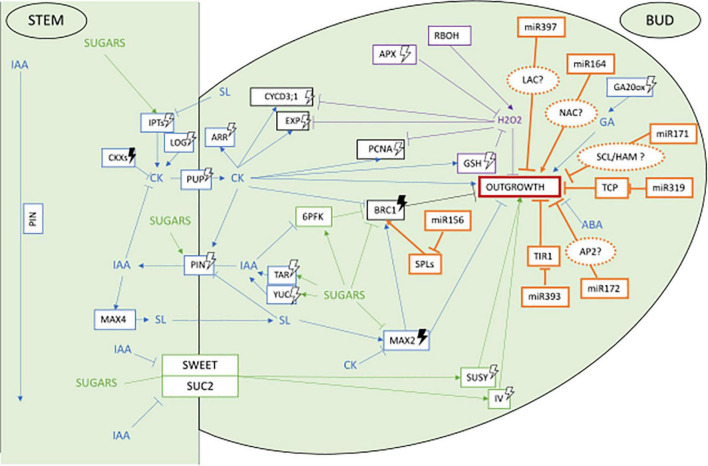
Network of main actors controlling axillary bud outgrowth in the rosebush Rosa ‘Radrazz’* and the potential targets of miRNAs based on literature in other plant species** Potential gene targets of these miRNAs in the control of bud outgrowth are represented in dotted orange circles. miRNAs involved in control of bud outgrowth according to the literature are represented in orange. Blue represents hormonal pathway, green sugars pathway, purple ROS pathway. Arrows head means induction, straight lines mean repression, white lightning bolt means induction by light, darked lightning bolts means induction by darkness. *Girault et al. (2008), Girault et al. (2010), Henry et al. (2011), Choubane et al. (2012), Rabot et al. (2012), Rabot et al. (2014), Djennane et al. (2014), Barbier et al. (2015), Roman et al. (2016), Roman et al. (2017), Porcher et al. (2020), Porcher et al. (2021), Wang M. et al. (2021). **Jiao et al. (2010), Miura et al. (2010), Wang et al. (2010), Wei et al. (2012), Xia et al. (2012), Curaba et al. (2013), Zhou et al. (2013), Zhang et al. (2013), Aung et al. (2015), Wang L. et al. (2015), Wang Y. et al. (2015), Huang et al. (2017), Kravchik et al. (2019), Sun et al. (2019), Cui et al. (2020), Lian et al. (2021), Wang R. et al. (2021), Zhan et al. (2021).

Several reviews have recently described in depth the current knowledge on the processes controlling axillary bud outgrowth in plants and their control by light (Leduc et al., 2014; Schneider et al., 2019; Wang M. et al., 2020; Kotov et al., 2021). As a pre-requisite to discuss their possible regulation by miRNAs, we provide here a brief overview of the main actors involved in the photocontrol of axillary bud outgrowth with a particular focus on rosebush. Main actors and their interactions are presented in Figure 1.

### Main Actors and Their Roles in the Control of Axillary Bud Outgrowth

Axillary bud outgrowth is controlled by a complex interplay between several main actors such as hormones, nutrients in particular sugars and ROS (Figure 1).

Concerning hormones, strong interactions between auxin (IAA), cytokinins (CK), and strigolactones (SL) form a core regulatory hormonal network instrumental for the release of apical dominance and induction of axillary bud outgrowth (Domagalska and Leyser, 2011; Barbier et al., 2019, Figure 1, blue lines). A first proposed mechanism suggests IAA inhibits axillary bud outgrowth indirectly through repression of CK synthesis and promotion of SL synthesis and signaling, both hormones being able to enter axillary buds and to act directly on axillary buds (Beveridge et al., 2000; Brewer et al., 2009; Ferguson and Beveridge, 2009). The balance of their opposing effects (CK promoting and SL repressing bud growth) would determine the fate (paradormancy vs. outgrowth) of the axillary bud. Through a second suggested mechanism, the basipetal flux of IAA produced by the apical meristem would also cause inhibition of axillary bud outgrowth by acting on the polarized distribution of the IAA efflux transporters PIN-FORMED (PIN) proteins in the axillary bud and by preventing it to export its own auxin (Bennett et al., 2006; Prusinkiewicz et al., 2009). In addition, a repressive role of ABA on axillary bud outgrowth was demonstrated in several species such as tomato, bean, arabidopsis, and rosebush (Tucker, 1976; Knox and Wareing, 1984; Gocal et al., 1991; Le Bris et al., 1999; Chatfield et al., 2000; Corot et al., 2017). In rosebush, a continued *de novo* synthesis of ABA contributes to maintaining bud endodormancy through control of cell cycle arrest in G2 phase (Le Bris et al., 1999). Application of ABA on the stem of rosebush and of other species also inhibits outgrowth of non-endodormant axillary buds nearby (Chatfield et al., 2000; Cline and Oh, 2006; Corot et al., 2017) while beheading of the main shoot causes a decrease in ABA contents in axillary buds along with their outgrowth (Knox and Wareing, 1984; Gocal et al., 1991). Last, gibberellins (GA) are hormones that control meristem cell differentiation in leaf primordia and internode elongation, both processes taking place during axillary bud outgrowth (Koornneef and van der Veen, 1980; Dodsworth, 2009). In rosebush, GA synthesis under the control of *RoGA20-oxidase* (*RoGA20ox*) and *RoGA3ox* and reduction of GA catabolism through down-regulation of *RoGA2ox* were shown to contribute to axillary bud outgrowth (Choubane et al., 2012).

As providers of energy, osmolytes and cell wall material, sugars are other key players in axillary bud outgrowth control (Bonicel et al., 1987; Marquat et al., 1999; Le Hir et al., 2006, Figure 1, green lines). In the rosebush, pea and sorgho, defoliation, decapitation or *in vitro* experiments showed axillary bud outgrowth is dependent on sucrose availability (Girault et al., 2010; Henry et al., 2011; Kebrom et al., 2012; Rabot et al., 2012; Mason et al., 2014). In rosebush *Rosa* ‘Radrazz’, *SUCROSE TRANSPORTER 2* (*RhSUC2*) and *RhSWEET10* control sucrose transport to the bud (Henry et al., 2011; Roman et al., 2016) where *RhSUSY* and *VACUOLAR INVERTASE 1* (*RhVI1*) catabolize sucrose into fructose and glucose for growth (Girault et al., 2010; Rabot et al., 2014). Promoting effects of non-metabolizable sucrose analogs on axillary bud outgrowth in rosebush suggests that sucrose may also play a signaling role during axillary bud outgrowth (Rabot et al., 2012; Barbier et al., 2015). Sucrose interacts with the hormonal control of axillary bud outgrowth (Barbier et al., 2015). Using detached rosebush buds *in vitro*, Barbier et al. (2015) showed that sucrose upregulates auxin synthesis [*TRYPTOPHAN AMINOTRANSFERASE RELATED 1* (*RhTAR1*), *YUCCA 1* (*RhYUC1*)] and auxin efflux [*PIN-FORMED 1* (*RhPIN1*), *SERINE/THREONINE PROTEIN PHOSPHATASE 2A* (*RhPP2A*), *PINOID* (*RhPID*)] genes in buds and represses strigolactones *MORE AXILLARY BRANCHES 2* (*RwMAX2*) signaling gene. Promoting effect of sucrose onto axillary bud outgrowth also relies on repression of a main negative regulator of axillary bud outgrowth *BRANCHED1* (*BRC1*) (Aguilar-Martínez et al., 2007; Finlayson et al., 2010; Dun et al., 2012; Brewer, 2015; Wang et al., 2019a) by sucrose (Barbier et al., 2015; Wang M. et al., 2021).

Evidence is also repetitively brought showing that the oxidative metabolism contributes to the control of axillary bud outgrowth (Figure 1, purple lines). In grape, for example, application of sodium azide or hydrogen cyanamide or heat shock on endodormant buds represses *CATALASE* (*CAT*) scavenging activity, causing accumulation of hydrogen peroxide (H_2_O_2_) that promotes bud outgrowth (Or et al., 2002; Ophir et al., 2009; Pérez et al., 2009; Vergara et al., 2012; Sudawan et al., 2016; Meitha et al., 2018). In non-endodormant buds, H_2_O_2_ appears to play an opposite role. Hence, in tomato, *rboh* mutants in which the capacity to produce apoplastic H_2_O_2_ is altered, a highly branched phenotype is observed suggesting that H_2_O_2_ contributes to axillary bud outgrowth inhibition (Sagi et al., 2004; Chen et al., 2016). In non-endodormant rosebush axillary buds subjected to apical dominance, a high content of H_2_O_2_ is present and contributes to bud arrest. Upon stem decapitation, activation of ROS scavenging activity is observed that causes a rapid decrease in H_2_O_2_ content along with axillary bud outgrowth (Porcher et al., 2020). In rosebush buds, scavenging activity is mainly due to the ascorbate–glutathione cycle (AsA–GSH). Transcription of glutathione biosynthesis genes *RhGSH1* as *RhGSH2* as well as of ascorbate peroxidase *RhAPX1* and glutathione reductase *RhGR1* are indeed actively transcribed in axillary buds after plant beheading and during axillary bud outgrowth while catalase *RhCAT* is not. These up-regulations are followed by increased corresponding enzymatic activities (Porcher et al., 2020).

Suppression of bud dormancies allows axillary bud outgrowth characterized by resumption of DNA replication and cell cycle (Shimizu and Mori, 1998; González-Grandío et al., 2013). Increased transcriptional activity of *PROLIFERATING CELL NUCLEAR ANTIGEN* (*PCNA*), *CELL DIVISION CYCLE 2* (*CDC2*), *CYCLIN B* (*CYCB*, Devitt and Stafstrom, 1995), and *CYCLIN D3* (*CYCD3*) takes for example rapidly place in pea (Shimizu and Mori, 1998), apple trees (Li et al., 2018) and in *Rosa* buds (Roman et al., 2016; Porcher et al., 2020) upon dormancy break. Cell division along with cell wall expansion contribute to growth of organ primordia and lead to protrusion of young leaves out of buds scales. In rosebush, up-regulation of *EXPANSINS RhEXPA1, 2*, and *3* take place in axillary buds within a few hours after stem decapitation and is promoted by CK (Roman et al., 2016, 2017).

### Light Regulation of Main Actors of Axillary Bud Outgrowth

Spectral quality and light intensity both impact axillary bud outgrowth in a range of herbaceous and perennial species (Leduc et al., 2014; Demotes-Mainard et al., 2016; Huché-Thélier et al., 2016). Unlike other species such as Arabidopsis, tomato, and poplar (Girault et al., 2008), axillary buds of rosebush have an absolute need for light to grow out (Girault et al., 2008). Total inhibition of axillary bud outgrowth under darkness and promotion by light offers a great opportunity to examine the light control of bud outgrowth in this species and to deepen our knowledge of this ecodormancy in plants (Leduc et al., 2014; Demotes-Mainard et al., 2016; Huché-Thélier et al., 2016; Bertheloot et al., 2020).

In the rosebush, red (R) and blue (B) lights promote axillary bud outgrowth while far-red (FR) light is inhibitory (Girault et al., 2008). These responses are consistent with the shade avoidance syndrome of plants. In rosebush, monochromatic blue or red light alone is also able to trigger and sustain all processes involved in axillary bud outgrowth, suggesting cross links and redundancy in the transduction pathways of R and B lights (Abidi et al., 2013). The acrotonic outgrowth pattern observed in rosebush can also be deeply modified by localized dark treatment along the rosebush stem (Djennane et al., 2014) or temporary exposure to low light prior to full light exposure (Demotes-Mainard et al., 2013), indicating fine spatial regulation of axillary bud outgrowth patterns along the stem by light.

Transcriptional control of light over hormones, sugars, ROS, cell division and growth was demonstrated in the rosebush using contrasted lighting conditions (darkness vs. white light, monochromatic vs. white light) and exogenous hormonal and sugar treatments. From these studies, demonstration was made that CK are initial targets of the light control pathway (Roman et al., 2016). More precisely, bud exposure to light triggers a rapid and strong upregulation of genes involved in CK synthesis [*ISOPENTENYLTRANSFERASE* (*RhIPT3*, *RhIPT5*)], activation [*LONELY GUY 8* (*RhLOG8*)], and transport [*PURINE PERMEASE 5* (*RhPUP5*)] and to the repression of CK catabolism gene *CYTOKININ OXIDASE/DEHYDROGENASE 1* (*RhCKX1*). This leads to CK accumulation in nodes and buds which then causes an up-regulation of IAA biosynthesis and transport (*RhYUC1*, *RhPIN1*), sugar (*RhSUSY*, *RhVI*, *RhSUC2*, and *RhSWEET10*) synthesis and transport genes, and repression of axillary bud outgrowth inhibitory genes (*RhBRC1* and SL signaling *RwMAX2*). CK upregulation during light control of rosebush axillary bud outgrowth also acts through ROS scavenging (Porcher et al., 2021). In particular, the AsA-GSH pathway is activated by light and CK, causing a decrease in H_2_O_2_ content in bud (Porcher et al., 2021). These light regulations finally lead to axillary bud outgrowth through activation of cell cycle *RhPCNA*, and cell expansion *RhEXPA* genes (Roman et al., 2016, 2017). Additionally, in response to reduced light intensity, Corot et al. (2017) showed that inhibition of axillary bud outgrowth in the rosebush is associated with reduced levels of CK and increased levels of ABA. Exogenous delivery of CK and ABA to the stem confirmed their antagonistic action in the control of axillary bud outgrowth by light intensity (Corot et al., 2017). Light regulation of GA also contributes to the photocontrol of axillary bud outgrowth in the rosebush through upregulation of the expressions of biosynthesis genes *GA20ox* and *GA3ox* and repression of catabolic gene *GA2ox* (Choubane et al., 2012).

In dark, conversely, repression of axillary bud outgrowth results from up-regulation of repressor genes such as *RhBRC1* and SL signaling *RwMAX2* as well as repression of ROS scavenging AsA-GSH pathway (Djennane et al., 2014; Roman et al., 2016; Porcher et al., 2021). Repression of sucrose transport and catabolism under darkness leads to a switch toward sorbitol metabolism, seemingly as a survival mechanism. Strong upregulation of *NAD-DEPENDANT SORBITOL DEHYDROGENASE* (*RhNAD-SDH*) transcriptional activity is indeed observed upon exposure of buds to darkness (Girault et al., 2010).

## Extending Our Knowledge of Conserved miRNAs and Their Regulation of Axillary Bud Outgrowth to Rosebush

Beside the regulatory pathways discussed above, the role of miRNAs in bud outgrowth control was more recently discovered. Numerous miRNAs showing high degree of similarity in their sequence were found in the genomes of many plant species and thus defined as conserved miRNAs (Weber, 2004; Zhang et al., 2006). Some of these were identified as key players in axillary bud outgrowth. Yet, the role of these miRNAs has been explored only in a few model species such as Arabidopsis, maize, and rice, the two latter concentrating on a grass specific branching process, called tillering. Here, we provide an up-to-date summary of roles of conserved miRNAs in branching and provide new data of the putative role of these conserved miRNAs in rosebush. Branching phenotypes of miRNAs and target gene-resistant mutants are summarized in Table 1.

**TABLE 1:** Main conserved miRNA families and their associated targets causing shoot branching modulation.

miRNA families	Known target	Species	Plants	References
156	SPL	Rice, Lotus, Medicago, Soya, Arabidopsis	MiR-OE: Increased branching	Wei et al., 2012; Aung et al., 2015; Wang L. et al., 2015; Wang Y. et al., 2015; Sun et al., 2019
		Tomato	TG-LF: Increased branching	Cui et al., 2020
		Rice	TG-MR: reduced branching	Jiao et al., 2010; Miura et al., 2010
164	NAC	Arabidopsis, Cotton	MiR-OE: Increased branching TG-MR: Reduced branching	Zhan et al., 2021
171	GRAS/SCL/HAM	Arabidopsis, Barley	MiR-OE: Reduced branching	Wang et al., 2010; Curaba et al., 2013
		Tomato	MiR-LF: Increased branching	Schulze et al., 2010; Wang et al., 2010; Kravchik et al., 2019
		Arabidopsis	TG-LF: Reduced shoot branching	Huang et al., 2017
		Tomato	TG-OE: Increased branching	Wang et al., 2010
		Arabidopsis	TG-MR: Increased branching	
172	AP2	Arabidopsis	MiR-LF: Increased branching	Lian et al., 2021
319	TCP	Bentgrass	MiR-OE: Decreased branching	Zhou et al., 2013
		Rice	MiR-LF: Increased branching	Wang R. et al., 2021
393	TIR1/AFB	Rice	MiR-OE: Increased branching	Xia et al., 2012
397	LAC	Rice	MiR-OE: Decreased branching	Zhang et al., 2013
444	MADS	Rice	MiR-OE: Reduced branching TG-OE: Increased branching TG-MR: Increased branching	Guo et al., 2013
529	SPL	Rice	MiR-OE: Increased tillering MiR-LF: Reduced branching	Yan et al., 2021

*MiR-OE, miRNA overexpression phenotype; MiR-LF, miRNA loss-of-function or knock-down phenotype; TG-MR: target resistant phenotype; TG-LF, target loss-of-function or knock-down phenotype; TG-OE, target overexpression phenotype.*

The study of tillering in rice (*Oryza sativa*) has been instrumental in uncovering the role of miRNAs in bud outgrowth. To-date, miR156 is one of the best characterized miRNA families. MiR156 plays a major role in regulating shoot branching by targeting a subset of the *SQUAMOSA PROTEIN-LIKE* (*SPLs*) genes. SPL transcription factors are found in all green plants and are involved in many developmental regulatory processes such as phase transition and flowering, plastochron control, leaf development, fruit ripening and response to stresses (Wang and Wang, 2015). *SPL* genes are ordered into nine clades, of which six are regulated by miR156 (Preston and Hileman, 2013). In rice, increased expression level of *OsSPL14* resulting from a mutation in its miR156 binding site leads to reduced branching from vegetative buds (Jiao et al., 2010; Miura et al., 2010). Interestingly, this mutation has opposite effects on branching during the reproductive phase, as it leads to increased inflorescence (panicle) branching and altogether forms the molecular basis of a quantitative locus known as *WEALTHY FARMER’S PANICLE* or *IDEAL PLANT ARCHITECTURE 1 (IPA1)* that improves rice productivity. On the contrary, high miR156 levels increase tiller formation in the dominant *Corngrass1* maize mutant (Chuck et al., 2007). An increased branching resulting from miR156 overexpression was also reported in other species including Arabidopsis (Wei et al., 2012; Tian et al., 2014), alfalfa (Aung et al., 2015), lotus (Wang Y. et al., 2015), soya (Sun et al., 2019), and tomato. In this last species, the increased branching phenotype can be reverted by expression of a miR156-resistant *SPL13* gene (Cui et al., 2020). Modulation of miR156/SPL interaction affects branching through both initiation and outgrowth of the axillary buds (Wang L. et al., 2015). At least three pathways have been associated with such increased branching. First, *UNBRANCHED3*, the maize ortholog of *OsSPL14*, directly targets and represses the expression of cytokinin biosynthesis and signaling genes while promoting expression of cytokinin degradation genes, thus resulting in lower cytokinin levels and signaling (Du et al., 2017). Second, *OsSPL7*, another target of miR156 in rice, represses the expression of *OsGH3.8* which codes for an enzyme conjugating auxin to aspartate (Dai Z. et al., 2018). Hence, miR156 may affect auxin/cytokinin balance that is important for both formation and growth of the axillary meristems. Last, *OsSPL14* directly promotes the expression of *OsTB1*, the rice homolog of bud outgrowth repressor *AtBRC1* (Lu et al., 2013). In this last case, miR156 repression of *OsSPL14* would reduce *AtBRC1* inhibitory effect on bud outgrowth.

The study of tillering in rice has revealed additional miRNAs that contribute to the regulation of branching in this species (Yue et al., 2017). MiR529 that partially overlaps with miR156 also targets a subset of *SPL* genes. MiR529 is found in monocots but was specifically lost in some dicots (Morea et al., 2016). Recently, Yan et al. (2021) showed that modulation of miR529 activity increases rice tillering in a similar way as miR156.

Increased tillering was also observed in rice lines overexpressing miR393. This miRNA targets genes coding for auxin receptors [*TRANSPORT INHIBITOR RESPONSE 1* (*OsTIR1*) and *AUXIN SIGNALING F-BOX 2* (*OsAFB2*)]. Accordingly, rice lines overexpressing miR393 show a reduced response to auxin and lower expression of the *OsTB1* gene, which may account for increased axillary bud growth (Li et al., 2016).

Modified auxin signaling is also observed in rice plants expressing a miR160-resistant version of the *AUXIN RESPONSE FACTOR* (*OsARF18)* that develop less tillers (Huang et al., 2016).

While overexpression of miR156, miR529 or miR393 increases rice tillering, overexpression of the monocot-specific miR444 has an opposite effect (Guo et al., 2013). miR444 targets *OsMADS57* which is expressed in developing buds where *OsMADS57* represses expression of *D14* gene coding for the SL receptor. Hence, miR444 overexpression may lead to increased SL response and thus inhibition of bud outgrowth.

Finally, overexpression of miR397 that targets rice *OsLAC*, a laccase polymerizing monolignols into lignin (Berthet et al., 2011; Zhao et al., 2013) also leads to a slight reduction in rice tiller number (Zhang et al., 2013).

Study in *Arabidopsis thaliana* also revealed the potential role of three other miRNAs in the control of branching. For example, miR171 was shown to target genes coding for the GRAS transcription factors *SCARECROW-LIKE SCL6-II*, *SCL6-III*, and *SCL6-IV* [also named *LOST MERISTEMS1-3* (*LOM*) or *HAIRY MERISTEM* (*HAM*)] (Schulze et al., 2010; Wang et al., 2010). These proteins are required for proper meristem function, including axillary meristem formation, and act by promoting the specification of the stem cells in the apical region of the meristem (Zhou et al., 2018; Han et al., 2020). Arabidopsis lines overexpressing miR171 show a massive reduction in the number of secondary branches, which is reverted by the expression of a miR171-resistant version of any of the 3 *HAM* genes (Wang et al., 2010). In tomato also, reduced miR171 activity or overexpression of the tomato *HAM* gene *SlGRAS24* leads to increased branching suggesting that miR171 represses *HAM* gene expression to limit branching in these species (Huang et al., 2017; Kravchik et al., 2019). *SlGRAS24* modulates auxin and gibberellin signaling, but whether it affects the formation of the axillary meristem, or its outgrowth is not known yet (Huang et al., 2017). The function of miR171 in shoot branching inhibition appears to be conserved in monocots, as overexpression of this miRNA reduces branching in barley (Curaba et al., 2013).

In Arabidopsis, miR164 targets a subset of the NAC genes, including *CUP-SHAPED COTYLEDON* (*CUC*) genes that are important for a wide range of meristem-related developmental processes. Recent studies performed in cotton and in the heterologous Arabidopsis system suggest that repression of *CUC2* by miR164 is important for branch outgrowth (Zhan et al., 2021). Specifically, *CUC2* interaction with *BRC1* may modulate ABA levels to control bud growth (Zhan et al., 2021).

An antagonistic role of miR172 and of miR156 in the regulation of Arabidopsis branching was also demonstrated (Wang et al., 2009). The quintuple mutant of the five *MIR172* genes indeed shows an increased branching as does a line overexpressing *MIR156b* (Wei et al., 2012; Lian et al., 2021). *MiR172a* and *d* are the *MIR172* genes playing a major role in the repression of branching in Arabidopsis (Lian et al., 2021).

Research in other less studied plant species has also brought additional knowledge on the role of other miRNAs in branching. For instance, in bentgrass (*Agrostis stolonifera*), overexpression of miR319 leads to a slight decrease in tillering (Zhou et al., 2013). MiR319 targets a subset of the *TEOSINTE BRANCHED1/CYCLOIDEA/PCF* (*TCP*) transcription factor family which are involved among others in the transition from cell proliferation to differentiation during plant development (Palatnik et al., 2003). In line with these data, recent work in rice shows that inactivation of miR319 correlates with increased expression of *OsTCP21* and increased number and length of tiller buds (Wang R. et al., 2021). In *Camellia sinensis*, the expression of *MIR319c* is significantly reduced during bud outgrowth while that of its target *TCP2* is significantly upregulated (Liu et al., 2019).

Altogether, it appears that several conserved miRNAs contribute to branching control. While in some cases, their effect could be precisely attributed to meristem initiation or to their later outgrowth (Wang L. et al., 2015), this has not been systematically analyzed (Huang et al., 2017; Lian et al., 2021). In addition, as some of these miRNA/target couples are involved in many processes, the effect on branching could be sometimes indirect. This may be the case for example for miR156 that affects the duration of the vegetative phase and therefore the number of nodes bearing axillary meristems.

As described in part 1, a lot of information has been gained on axillary bud outgrowth regulatory pathways and its photocontrol through study of the rosebush *Rosa* ‘Radrazz’. However, the role of miRNAs in rosebush axillary bud outgrowth has not yet been explored. As a first step toward this, we analyzed whether the conserved miRNAs described in Table 1 were also expressed in rosebush axillary buds and if they could interact with the same known targets. To answer these questions and since no annotation of the mature miRNA sequences is available on the reference *Rosa* genome (*Rosa chinensis*, Hibrand Saint-Oyant et al., 2018; Raymond et al., 2018), we performed a high throughput profiling of the miRNA components of *Rosa* ‘Radrazz’ axillary buds. To induce axillary bud outgrowth, stems were beheaded to release apical dominance and were grown in light. We collected buds samples right after beheading and 6 h after beheading. This time point was chosen as it was late enough after beheading to allow key molecular mechanisms of the outgrowth process and of the light control to take place (Barbier et al., 2015; Roman et al., 2016; Porcher et al., 2020, 2021; Method detailed in Supplementary Method I) and early enough to minimize feed-back loop regulations of miRNAs that could be activated following resumption of axillary meristem outgrowth. From the sequenced small RNAs, prediction and annotation of the *Rosa* mature miRNAs sequences were achieved using *Arabidopsis thaliana* miRNAs database^1^ (Supplementary Data 1). Annotated *Rosa* miRNAs sequences were next aligned against orthologs of the known target genes described in literature (Table 1) and found in *Rosa chinensis* genome using target prediction web server’s psRNAtarget (Dai X. et al., 2018).^2^ Together this allowed us to take a snapshot of miRNAs present in buds upon suppression of apical dominance and during an early phase of bud growth and then predict their targets in *Rosa* (Table 2).

**TABLE 2:** Conserved miRNAs expressed in *Rosa* ‘Radrazz’ buds and their predicted gene targets.

Conserved miRNA families	Total number of miRNA family members identified in *Rosa* ‘Radrazz’ buds and names of those presenting binding ability to known target identified in other plants species	Target genes identified in *Rosa chinensis* genome with an expectation ≤3.5.	*Rosa chinensis* homologs accession number (Hibrand Saint-Oyant et al., 2018)	*Arabidopsis thaliana* orthologs accession number (TAIR 10 release)
156	14	*SPL2*	RC4G0346800	AT5G43270
	miR156c/miR156f/ miR156c_1/miR156k_ 1/ miR156a/miR156a-5p/ miR156j_1/miR156k_2/ miR156_2/miR156g_1	*SPL3*	RC5G0081600	AT2G33810
		*SPL6A*	RC4G0415800	AT1G69170
		*SPL6B*	RC7G0063500	AT1G69170
		*SPL6C*	RC7G0063900	AT1G69170
		*SPL9*	RC3G0139500	AT2G42200
		*SPL10*	RC1G0282700	AT1G27370
		*SPL13A*	RC7G0120000	AT5G50570
		*SPL16A*	RC2G0684700	no SPL16 in
		*SPL16B*	RC4G0344900	Arabidopsis
164	7	*NAC1*	RC5G0238100	AT1G56010
	miR164a_2/miR164a_4/miR164b/164e-5p/ miR164f_1	*NAC100_1*	RC7G0049200	AT5G61430
		*NAC100_2*	RC7G0049800	AT5G61430
		*NAC100_3*	RC2G0616000	AT5G61430
		*CUC2*	RC0G0187100	AT5G53950
171	19	*SCL6*	RC1G0287300	AT4G00150
	miR171b/miR171b-3p/miR171b-3p_3/miR171f- 3p/miR171a-3p/miR171a-3p_1/miR171a_3/ miR171b_2/miR171b_ 3/miR171_2/miR171c_3/ miR171d_1/miR171f_3	*SCL6*	RC7G0240100	AT4G00150
172	17	*RAP*	RC1G0423500	AT2G28550
	miR172a_4/miR172i/miR172a_2/miR172a_3/ miR172c-3p/miR172d_2/miR172e-3p_1/ miR172a_1/miR172b/miR172e-3p/miR172g-3p/ miR172f	*2.7/TOE 1*	RC5G0530900	AT2G28550
		*RAP*	RC2G0197000	AT4G36920
		*2.7/TOE 1*	RC3G0243000	AT5G67180
		*AP2*		
		*TOE3*		
319	11	*TCP2*	RC5G0134300	AT4G18390
	miR319_1/miR319a_1/miR319a-3p/ miR319c_1/miR319f_1/miR319g/miR319a/ miR319b_1/miR319c_2	*TCP4*	RC5G0279600	AT3G15030
393	8	*AFB2*	RC2G0688700	AT3G26810
	miR393a_1/miR393a_3/miR393-5p/miR393a/ miR393a-5p/miR393b-5p/miR393h	*TIR1*	RC6G0421400	AT3G62980
397	3	*LAC2*	RC5G0566900	AT2G29130
	miR397-5p_1/miR397a_6/miR397a_3	*LAC3*	RC5G0617100	AT2G30210
		*LAC4A*	RC3G0283200	AT2G38080
		*LAC4B*	RC5G0590200	AT2G38080
		*LAC7A*	RC3G0278900	AT3G09220
		*LAC7B*	RC5G0655700	AT3G09220
		*LAC11A*	RC6G0173800	AT5G03260
		*LAC11B*	RC6G0174100	AT5G03260
		*LAC11C*	RC6G0176400	AT5G03260
		*LAC11D*	RC6G0176600	AT5G03260
		*LAC11E*	RC6G0177800	AT5G03260
		*LAC11F*	RC6G0181300	AT5G03260
		*LAC17A*	RC3G0261900	AT5G60020
		*LAC17B*	RC3G0262200	AT5G60020
		*LAC17C*	RC5G0533800	AT5G60020

*Conserved Rosa ‘Radrazz’ mature miRNAs were identified from small RNA high throughput sequencing of axillary buds upon beheading and 6 h after beheading under light conditions. Target prediction was achieved using the web server’s psRNAtarget (Dai X. et al., 2018; https://www.zhaolab.org/psRNATarget/) by using an expectation ≤3.5 to avoid false positive results. For target genes, corresponding accession numbers of Rosa chinensis homologs and Arabidopsis thaliana orthologs are indicated.*

Our bioinformatic analysis shows that out of the 9 miRNAs families previously described as involved in the control of bud outgrowth (as mentioned above), 7 were found in *Rosa* (Table 2). The two missing families, miR444 and mi529, are also missing in other dicots. This result reinforces the hypothesis that miR529 was lost in eudicots during evolution and that miR444 is only present in monocots (Sunkar et al., 2005; Ortiz-Morea et al., 2013). Each *Rosa* miRNA family is represented by several members (i.e., 14 in miR156 family; 7 in miR164 family). Similar numbers of members were found in Arabidopsis except for miR171, 172, and 319 families in which a much lower number of members were identified in *Arabidopsis* (6, 9, and 3 members, respectively) in comparison to *Rosa*. This may suggest differences in the tuning of the regulations controlled by these miRNA families between *Rosa* and *Arabidopsis*.

Binding ability of *Rosa* miRNAs to known targets was predicted by searching sequences complementary to the miRNAs in the putative targets. Same target gene families were found in the *Rosa* genome as described for other species (Table 1). These findings suggest that regulations involving these miRNA families and their associated targets may also be conserved in *Rosa* axillary buds.

Bioinformatic analysis allowed identification of several members of each target gene family in *Rosa* (Table 2). Further experimental investigations are needed for confirmation of their interaction.

Results are summed up in orange in Figure 1 showing the network of main actors controlling axillary bud outgrowth in the rosebush Rosa ‘Radrazz’ and the potential roles of miRNAs based on literature in other plant species and the miRNA/target pairs we identified here.

## Crosstalk Between Light Signaling Pathway and miRNAs Encoding Gene Expression

Light is a major environmental cue that controls bud outgrowth in many plant species and light regulation of axillary bud outgrowth at transcriptional level has been well documented in rosebush (Girault et al., 2008; Rabot et al., 2014; Roman et al., 2016; Porcher et al., 2020). Yet, little is known to-date on the involvement of miRNAs in light-mediated axillary bud outgrowth mechanisms. Therefore, we summarize here the present knowledge on light effects on the miRNA pathway in general, affecting for instance the stability or functionality of the core proteins involved in the maturation of miRNAs (Sánchez-Retuerta et al., 2018; Park et al., 2021). In addition to such a general effect on the whole miRNA pathway, light also affects the expression of some *MiRNA* genes specifically. Red (R) and Blue (B) light signal transduction engages photoreceptors, including phytochromes (PHY) and cryptochromes (CRY) and their downstream effectors as *ELONGATED HYPOCOTYL 5* (*HY5*) and *PHYTOCHROME INTERACTING FACTORS* (*PIF4*). *PHYB* plays a main role in the photocontrol of bud outgrowth (Kebrom and Mullet, 2016). Recently, Holalu et al. (2020) reported that PIF4 and PIF5 contribute to the suppression of branching resulting from *phyB* loss-of-function and a low R/FR ratio. This phenotype is correlated to *BRC1* expression induction by PIF4/PIF5 and to abscisic acid (ABA) accumulation in axillary buds. Interestingly, several reports indicated that modulation of miRNA expression may contribute to the PHY-mediated light response, raising the hypothesis that this may contribute to branching modulation. For instance, analysis of miRNAs and their PHYB-mediated targets in rice leaves identified a total of 135 miRNAs differentially expressed between the WT and *phyB* mutant (Sun et al., 2015). This finding suggests that these miRNAs are directly or indirectly controlled by PHYB and participate in PHYB-mediated light signaling. In the same line, HY5 and PIFs transcription factors were reported to directly control the expression of several miRNA genes (Zhang et al., 2011; Xie et al., 2017). More recently, in Arabidopsis, PIF4 was reported to promote expression of genes encoding miR156/157, miR160, miR165/166, miR167, miR170/171, and miR394 and to reduce the expression of the genes encoding miR172 and miR319 by binding to the promoters of these miRNA genes (Sun et al., 2018). Compared to wild type, corresponding miRNA mutants had altered hypocotyl phenotypes, supporting a role for specific miRNAs in photomorphogenesis (Sun et al., 2018). In the extremophile plant *Eutrema salsugineum* under long-term action of R, Pashkovskiy et al. (2021) reported an increase in the expression of *PHYA*, *PHYB*, and *PHYC* as well as of *PIF4* and *PIF5* together with that of miR395, miR408 and miR165. In addition, they also observed a decrease in *HY5*, *miR171*, *miR157*, and *miR827* expressions. These data suggest that the quantity of these miRNAs in *E. salsugineum* is light-regulated in a PHY and PIF-mediated manner. Also, in *Solanum tuberosum* leaves, R light can induce miR398, 399, 408, 482, 8036, and 8049 expressions (Qiao et al., 2017). Among miRNAs whose expression is regulated by light, some (miRNA156, 171, 172, and 319) are involved in branching control (Table 1). These observations thus indicate that light *via* a PHY-dependent pathway may control bud outgrowth through modifying the expression of several miRNAs.

A key event in bud outgrowth is the initiation and expansion of leaves. While no experimental data report direct interaction between light signaling and miRNAs biogenesis in the control of leaf organogenesis during bud outgrowth, some works demonstrate that light regulates leaf expansion (Romanowski et al., 2021), and this partly occurs through miRNAs regulation (Pashkovskiy et al., 2016). Hence, Pashkovskiy et al. (2016) reported that blue light causes a significant increase in *miR167* expression which decreases the level of auxin-dependent *ARF6* transcripts in *Arabidopsis* leaves, thereby allowing leaf expansion. ARF transcription factors play an important role in the auxin-mediated gene transcription pathway by interacting with Aux/IAAs proteins, a key pathway involved in bud outgrowth (Vanneste and Friml, 2009; Dierck et al., 2016). In the same line, blue light (B) specifically down-regulates miR156 and miR157 and upregulates their target genes *SPL9* and *SPL15* during *Brassica rapa* subsp. Rapa seedling development (Zhou et al., 2016). These results, combined to increase shoot branching phenotype caused by *miR156* overexpression in *Brassica napus* (Wei et al., 2010), support the hypothesis of a crosstalk between light signaling and miRNAs regulation and suggest miR156/SPL couple acts as regulatory hub of bud outgrowth in response to light. More recently, Dong et al. (2020), found 20 miRNAs differentially expressed in tomato leaves after blue light treatment. Among them, *sly-miR9472-3p* expression is up-regulated by blue light treatment and is negatively correlated to the expression of its target gene *IPT5*, a cytokinin biosynthesis gene which plays a crucial role in promoting axillary bud outgrowth (Barbier et al., 2015; Roman et al., 2016). All these data suggest that miRNAs may well be involved in the light control of leaf expansion during bud outgrowth.

## Bioinformatic Analysis of Putative New miRNAs in the Control and Photocontrol of Bud Outgrowth in *Rosa*

As described above, rosebush is a pertinent model to understand how light controls axillary bud outgrowth. Since little has been described on the role of miRNAs in the photocontrol of axillary bud outgrowth, we took advantage of the rose model to further examine whether components of the axillary bud outgrowth regulatory network (Figure 1) may be targets of post-transcriptional regulation *via* miRNAs.

To address this question, full length sequences of genes from this network were identified on *Rosa chinensis* genomes (Hibrand Saint-Oyant et al., 2018; Raymond et al., 2018) and confronted against the mature miRNAs sequences we identified in *Rosa* axillary bud from small RNA-seq using target prediction web server’s psRNAtarget (Dai X. et al., 2018; see text footnote 2) (Supplementary Table 1).

Our bioinformatic analysis reveals that 9 out of the sixty-five genes related to axillary bud outgrowth and its photocontrol in *Rosa* are predicted to be targeted by 7 miRNA families. These families are conserved in plants. Out of these 7 miRNAs, only miR156 and miR164 have already been described as involved in control of bud outgrowth (Table 1). On the contrary, five miRNAs (miR159, miR166, miR399, miR477, and miR8175) were not yet identified for a possible role in the control of bud outgrowth but were characterized for other functions. This could be either because their contribution to axillary bud outgrowth could be masked by the action of other more prominent miRNAs or, alternatively, because the strict light dependence of rosebush axillary bud outgrowth involves novel regulatory circuits that are less conserved in other species, and thus less characterized. MiR159 has been reported in growth transition, programmed cell death, and seed germination (Reyes and Chua, 2007; Alonso-Peral et al., 2010; Guo et al., 2017). MiR166 is implicated in different developmental processes such as regulation of shoot meristem development (Zhang and Zhang, 2012), root growth and development (Boualem et al., 2008; Singh et al., 2014) and seed maturation (Tang et al., 2012). MiR399 has been studied for its role in phosphate homeostasis and starvation (Baek et al., 2013; Tian et al., 2018). For the two last, miR477 and miR8175, to date little data is available concerning their biological function. MiR477 is implicated in plant immunity in *Camellia sinensis* and in *Gossypium hirsutum* (Hu et al., 2020; Wang S. et al., 2020), while miR8175 has been studied in *Arabidopsis thaliana* roots, and found to be upregulated by high-light intensity (Anna et al., 2019).

Among the nine genes identified as predicted targets, three genes participate in hormone signaling including two genes involved in the cytokinin pathway (*RhCKX1*, *RhCKX6*) both targeted by *miR159a_1* and one in the strigolactones pathway (*RwMAX4*) targeted by six miR166 members. These genes are involved in the repression of bud outgrowth in *Rosa*. Interestingly, none of the genes involved in auxin and gibberellin pathways are found as potential targets of post-transcriptional regulation *via* miRNAs. Concerning sugar control, two genes (*RhSUSY1*, *Rh6PFK*) are targeted by two different miRNAs: *miR8175* and *miR166e-5p*, respectively. Besides hormonal and sugar control, two genes involved in ROS metabolism are potential targets of miRNAs. The first one, *RhAPX1*, encoding antioxidant enzyme is targeted by three miR164 isoforms (miR164a_4, 164b, and 164e-5p), the other one (*RhRBOHB1*) encoding a NADPH oxidase is targeted by *miR399e_5*. Finally, two genes involved in the cell division (*RhCYCD3*;2) and expansion (*RhEXPA3*) are identified as potential targets of miR477e and miR156j_1, respectively. The validation of the interaction between these 7 miRNAs and the predicted rosebush targets awaits experimental confirmation. It is however noteworthy that, in agreement with our findings, miR159 was previously shown as targeting CKX with negative correlation of their expression in response to pathogens in poplar (Zhao et al., 2012). Furthermore, suppressing miR159 leads to a decreased expression of the CK synthesis gene, *OsIPT*, of CK signaling genes *OsRR* and of the CK degradation genes *OsCKX* in rice (Zhao et al., 2017). Taken together, these data suggest that miR159 may play a role in cytokinin metabolism and signaling and support our prediction of miR159 targeting two *CKX* in rosebush during bud outgrowth.

**FIGURE 2:**
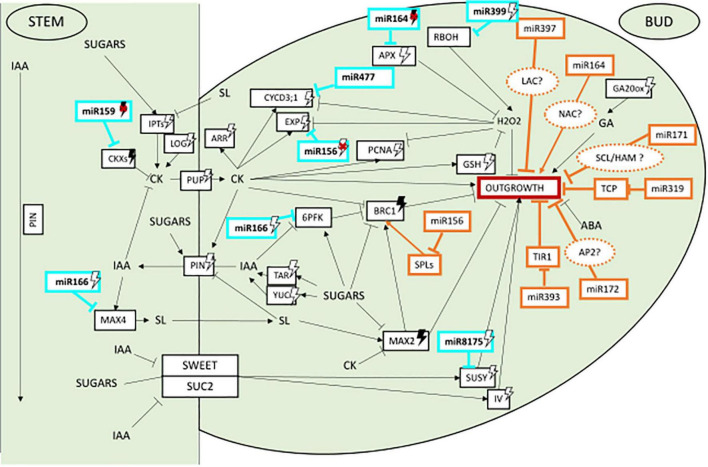
Potential role of miRNAs in the control of axillary bud outgrowth and their light regulation in plants. In bright blue miRNAs identified in the present study as putative novel regulators in control of bud outgrowth. Black arrows heads mean induction, straight lines mean repression, white lightning bolt means induction by light, darked lightning bolt means induction by darkness, red cross means repression of the miRNA.

Our study reveals that even though few genes of the regulatory network of axillary bud outgrowth appear under miRNA regulation, most of the pathways identified in *Rosa* are potential targets of miRNA regulation (Figure 2). This suggests that miRNAs act on multiple pathways of the complex network regulating bud outgrowth in rosebush. Interestingly, six of the nine identified genes targeted by miRNAs have a light regulated expression (Supplementary Table 1). This brings up the hypothesis that their respective associated miRNAs might potentially act in the light transduction pathway (Figure 2). Further studies addressing the light regulation of these miRNAs in rosebush will be required to progress in the understanding of their role during axillary bud outgrowth.

The other two miRNAs identified here, miR156 and miR164 were already described to play a role in regulating shoot branching (as mentioned above). While miR156j_1 *Rosa* isoform and the 3 miR164 *Rosa* isoforms are predicted to bind to their conserved targets *SPL* and *NAC* (Laufs et al., 2004; Wang and Wang, 2015; Blein and Laufs, 2016; Xu et al., 2016), new targets were also identified by our analysis: *EXPA3* and *APX1*, respectively, both genes being light-regulated during bud outgrowth in *Rosa* (Supplementary Table 1). The Arabidopsis orthologs of these Rosa targets (*AtEXP15* and *AtAPX1*) lack a binding site for the miRNA (data not shown). Therefore, it is attractive to speculate that the addition of these new targets may have appeared by evolution of their sequence to create a novel miRNA binding site. The resulting bifurcated pathway downstream the miRNA, with one conserved branch (*SPL* or *NAC*) and one branch more species-specific (*EXPA3* or *APX1*) could provide robustness to the effect of the miRNA on bud outgrowth.

## Conclusion and Future Perspectives

Multiple regulators have been described as acting in concert to fine tune, in an intricate network, axillary bud outgrowth as well as its photoregulation. While this network is mostly built on integrating knowledge gained at the transcriptional level, little is known to date about how post-transcriptional regulation fits into this network. Here, we provide new information by integrating post-transcriptional regulation of the photocontrol bud growth into this network. Based on the literature published mainly on Arabidopsis, rice and maize combined with our bioinformatic analysis on *Rosa* we explored specifically the potential role of miRNAs in the control of bud growth and how they may manage to interact with this network. Thus, we identified through bioinformatic analysis some conserved miRNAs families and their associated target genes as good candidates to play a potential role in the control of axillary bud outgrowth in the rosebush. Interestingly, these miRNAs have the ability to target key molecular players in all major pathways regulating bud outgrowth (including hormones, sugars, and ROS) with a far broader spectrum compared to previously reported studies. Additionally, according to literature, several of those such as miR156, miR164, miR166, and miR8175 are under light regulation which leads us to suggest that they may participate in the light-mediated mechanisms of axillary bud outgrowth. Whether or not these novel miRNA-gene target couples physically interact and demonstrate a biological relevance in the regulation of bud outgrowth and its photocontrol is still to be addressed. To this end, we need to move toward functional analysis and use of mutants.

Involvement of post-transcriptional factors in bud outgrowth control is not limited to miRNAs. Indeed, some studies have brought to light the possible role of small interfering RNAs (siRNAs) in this process (Ortiz-Morea et al., 2013; Kamthan et al., 2015). In this purpose, future research should be conducted to identify precisely which small RNAs are involved using deep sequencing and understand how they interact with miRNAs in the control of axillary bud outgrowth.

The understanding of the mechanisms behind miRNA control of bud outgrowth could be a great opportunity for improving agriculturally important traits such as plant architecture (Boumaza et al., 2010; Leduc et al., 2014; Huché-Thélier et al., 2016). Notably, it was shown recently that plant 5′ of primary miRNA contains a short open reading-frame (ORF) that encodes a peptide called micro-peptide (miPEPs). These miPEPs can raise the mature miRNA level by enhancing the transcription of their associated primary miRNA in a specific manner (Lauressergues et al., 2015). For that miPEPs could be used as powerful tools to lead to agronomic traits improvement under miRNA control (Couzigou et al., 2015).

## Data Availability Statement

The original contributions presented in the study are included in the article/Supplementary Material, further inquiries can be directed to the corresponding author/s.

## Author Contributions

JM, PL, NL, and JLG designed the experiments and wrote the manuscript. JM performed the experiments and bio informatic analysis. PL, NL, and JLG supervised the work. All authors read and approved the final manuscript.

## Conflict of Interest

The authors declare that the research was conducted in the absence of any commercial or financial relationships that could be construed as a potential conflict of interest.

## Publisher’s Note

All claims expressed in this article are solely those of the authors and do not necessarily represent those of their affiliated organizations, or those of the publisher, the editors and the reviewers. Any product that may be evaluated in this article, or claim that may be made by its manufacturer, is not guaranteed or endorsed by the publisher.

## Footnotes

1
http://www.mirbase.org/index.shtml

2
https://www.zhaolab.org/psRNATarget/
