# Supplementary material for: Photocontrol of Axillary Bud Outgrowth by MicroRNAs: Current State-of-the-Art and Novel Perspectives Gained From the Rosebush Model
Source: Front Plant Sci. 2022 Jan 31;12:770363. doi: 10.3389/fpls.2021.770363 (PMC8841825; doi:10.3389/fpls.2021.770363)
Supplement: Supplementary file 1 [file Table_1.docx]

Supplemental Table I: **Main genes involved in axillary bud outgrowth and its photocontrol in *Rosa* and predicted miRNAs after psRNAtarget analysis**. The corresponding accession numbers of the *Rosa chinensis* homologs and *Arabidopsis thaliana* orthologs are indicated. Light regulation of these genes in buds as reported in literature are mentioned as (a) Genes up-regulated by light, (b) Genes down-regulated by light, (c) Genes up-regulated by dark and (d) Genes down-regulated by dark.

| Biological processes pathways | Function | *Rosa* sp. genes described in axillary bud outgrowth control (Rh for *Rosa* ‘Radrazz’; Rw for *Rosa x wichurana*) | Light regulations | References | *Rosa chinensis* homologs accession number (Hibrand- Saint Oyant et al., 2018) | *Arabidopsis thaliana*  orthologs accession number (TAIR 10 release) | PsRNAtarget prediction results with an expectation ≤ 3.5. |
| --- | --- | --- | --- | --- | --- | --- | --- |
| Hormone - Auxin | Biosynthesis | *RhYUCCA1* | a,d | Barbier et al., 2015  Roman et al., 2016a, d | RC0G0230100 | AT4G32540 | not targeted |
|  |  | *RhTAR1* | a | Barbier et al., 2015  Roman et al., 2016 a | RC7G0149400 | AT1G23320 | not targeted |
|  | Transport | *RhPIN1* |  | Barbier et al., 2015 | RC7G0016500 | AT1G73590 | not targeted |
|  |  | *RhPID* |  | Barbier et al., 2015 | RC5G0142700 | AT2G34650 | not targeted |
|  |  | *RhPP2A* |  | Barbier et al., 2015 | RC4G0397100 | AT1G69960 | not targeted |
| Hormone - CK  Hormone - CK | Biosynthesis | *RhIPT3* | a | Barbier et al., 2015  Roman et al., 2016 a | RC6G0410100 | AT3G63110 | not targeted |
|  |  | *RhIPT5* | a | Barbier et al., 2015  Roman et al., 2016c | RC4G0245000 | AT5G19040 | not targeted |
|  | Activation | *RhLOG3* | a | Roman et al., 2016 a | RC6G0304400 | AT2G37210 | not targeted |
|  |  | *RhLOG8* | a | Roman et al., 2016a | RC6G0591500 | AT5G11950 | not targeted |
|  | Signaling | *RhARR3* | a | Roman et al., 2016 a | RC4G0477500 | AT1G59940 | not targeted |
|  |  | *RhARR5* | a | Roman et al., 2016 a | RC7G0031400 | AT3G48100 | not targeted |
|  | Transport | *RhENT1* | c | Roman et al., 2016 c | RC4G0469600 | AT1G70330 | not targeted |
|  |  | *RhPUP5* | a,d | Roman et al., 2016 a,d | RC2G0013600 | AT2G24220 | not targeted |
|  | Degradation | ***RhCKX1*** | **b,c** | **Barbier et al., 2015**  **Roman et al., 2016 b,c** | **RC3G0106400** | **AT2G41510** | **miR 159a_1** |
|  |  | ***RhCKX6*** | **b,c** | **Roman et al., 2016 b,c** | **RC1G0050300** | **AT3G63440** | **miR 159a_1** |
| Hormone - SL | Biosynthesis | *RwMAX1* |  | Djenanne et al., 2014 | RC6G0591100 | AT2G26170 | not targeted |
|  |  | *RwMAX3* |  | Djenanne et al., 2014 Barbier et al., 2015 | RC1G0278600 | AT2G44990 | not targeted |
|  |  | ***RwMAX4*** |  | **Djenanne et al., 2014 Barbier et al., 2015** | **RC6G0548900** | **AT4G32810** | **miR166/a/a-3p/e-3p/h-3p/m_2** |
|  | Signaling | *RwMAX2* | c | Djenanne et al., 2014 c | RC2G0386000 | AT2G42620 | not targeted |
| Hormone - GA | Biosynthesis | *RhGA20ox* | a | Choubane et al., 2012a | RC1G0530300 | AT4G25420 | not targeted |
|  |  | *RhGA2ox* | b,c | Choubane et al., 2012 b,c | RC5G0246800 | AT1G78440 | not targeted |
|  |  | *RhGA3ox* | a | Choubane et al., 2012a | RC2G0451600 | AT1G15550 | not targeted |
| Cell growth | Cell division | ***RhCYCD3;1*** | **a,d** | Roman et al., 2016 a,d | **RC2G0200400** | **AT4G34160** | **miR477e** |
|  |  | *RhPCNA1* | a,d | Roman et al., 2016 a,d | RC5G0585000 | AT1G07370 | not targeted |
|  | Cell wall expansion | *RhEXPA1* | a | Roman et al., 2017 a | RC5G0626300 | AT2G40610 | not targeted |
|  |  | *RhEXPA2* | a | Roman et al., 2017 a | RC3G0260200 | AT2G39700 | not targeted |
|  |  | ***RhEXPA3*** | **a,d** | **Roman et al., 2016 a,d**  **Roman et al., 2017** | **RC2G0618000** | **AT1G69530** | **miR156j_1** |
| Sugar | Sugar metabolism | ***RhSUSY1*** | **a,d** | **Roman et al., 2016a, d** | **RC2G0105700** | **AT5G20830** | **miR8175** |
|  |  | *RhNAD-SDH* | b,c | Girault et al., 2010b  Roman et al., 2016 c | RC1G0532900 | AT5G51970 | not targeted |
|  |  | *RhVI1* | a,d | Girault et al., 2010a  Roman et al., 2016 d | RC1G0516400 | AT1G62660 | not targeted |
|  |  | *RhVI2* | a | Rabot et al., 2012a | RC7G0234000 | AT1G12240 | not targeted |
|  |  | ***Rh6PFK*** |  | **Wang et al., 2021a** | **RC7G0357900** | **AT1G20950** | **miR166e-5p** |
|  |  | *RhPK* |  | Wang et al., 2021a | RC1G0556400 | AT5G52920 | not targeted |
|  |  | *RhMD* |  | Wang et al., 2021a | RC7G0239300 | AT4G17260 | not targeted |
|  |  | *Rh6PD* |  | Wang et al., 2021a | RC4G0422200 | AT3G29360 | not targeted |
|  |  | *RhG6PD* |  | Wang et al., 2021a | RC0G0007400 | AT5G13110 | not targeted |
|  |  | *Rh2OD* |  | Wang et al., 2021a | RC5G0355100 | AT4G26910 | not targeted |
|  |  | *RhHXK1* |  | Wang et al., 2021a | RC2G0068400 | AT4G29130 | not targeted |
|  | Transport | *RhSUC1* |  | Henry et al., 2011 | RC6G0482600 | AT1G71880 | not targeted |
|  |  | *RhSUC2* | a,d | Henry et al., 2011a | RC6G0239200 | AT1G22710 | not targeted |
|  |  | *RhSUC3* |  | Henry et al., 2011 | RC4G0436300 | AT2G02860 | not targeted |
|  |  | *RhSUC4* |  | Henry et al., 2011 | RC7G0494700 | AT1G09960 | not targeted |
|  |  | *RhSWEET10* | a | Roman et al., 2016 a | RC7G0116000 | AT5G50790 | not targeted |
|  |  | *RhSTP1* |  | Wang et al., 2021a | RC5G0057200 | AT1G11260 | not targeted |
| ROS | Cell redox homeostasis | *RhGR1* | a,d | Porcher et al., 2020a  Porcher et al.,2021 d | RC0G0077700 | AT3G24170 | not targeted |
|  |  | *RhGR2* | d | Porcher et al., 2020  Porcher et al.,2021 d | RC6G0391800 | AT3G54660 | not targeted |
|  | Detoxification | ***RhAPX1*** | **a** | **Porcher et al., 2020a** | **RC5G0530600** | **AT1G07890** | **miR164a_4 164b e-5p** |
|  |  | *RhAPX6* | a,d | Porcher et al., 2020a  Porcher et al.,2021 d | RC6G0464200 | AT4G32320 | not targeted |
|  |  | *RhCAT* | c | Porcher et al., 2020  Porcher et al.,2021 c | RC7G0342400 | AT4G35090 | not targeted |
|  | Glutathion biosynthesis | *RhGSH1* | a,d | Porcher et al., 2020a  Porcher et al.,2021 d | RC7G0192300 | AT4G23100 | not targeted |
|  | Glutathion biosynthesis | *RhGSH2* | a,d | Porcher et al., 2020a  Porcher et al.,2021 d | RC3G0364000 | AT5G27380 | not targeted |
|  | Respiratory Burst Oxidase Homologs | ***RhRBOHB1*** |  | **Porcher et al., 2021** | **RC3G0349300** | **AT1G09090** | **miR399e_5** |
|  |  | *RhRBOHB2* |  | Porcher et al., 2021 | RC3G0349100 | AT1G09090 | not targeted |
|  |  | *RhRBOHD* |  | Porcher et al., 2021d | RC4G0330300 | AT5G47910 | not targeted |
|  |  | *RhRBOHD2* |  | Porcher et al., 2021 | RC7G0236200 | AT5G47910 | not targeted |
|  |  | *RhRBOHD3* |  | Porcher et al., 2021d | RC7G0106000 | AT5G47910 | not targeted |
|  |  | *RhRBOHF* | b | Porcher et al., 2021d | RC7G0180700 | AT1G64060 | not targeted |
|  |  | *RhRBOHE1* |  | Porcher et al., 2021 | RC2G0105100 | AT1G19230 | not targeted |
|  |  | *RhRBOHE2* |  | Porcher et al., 2021 | RC2G0174800 | AT1G19230 | not targeted |
|  |  | *RhRBOHE3* |  | Porcher et al., 2021 | RC7G0059800 | AT1G19230 | not targeted |
|  |  | *RhRBOHHJ* |  | Porcher et al., 2021 | RC5G0534300 | AT5G60010 | not targeted |
|  | Monodehydroascorbic acid reductase | *RhMDHAR6* | a,d | Porcher et al., 2021a,d | RC7G0183000 | AT1G63940 | not targeted |
|  | Glutathion metabolic process | *RhDHAR1* | a,d | Porcher et al., 2021 | RC1G0433100 | AT1G75270 | not targeted |
|  | Integrator | *RhBRC1* | c | Barbier et al., 2015  Roman et al., 2016 c | RC7G0073800 | AT3G18550 | not targeted |
| Amino acid metabolism | Asparagine synthesis | *RhASN1* |  | Wang et al., 2021 | RC5G0605600 | AT3G47340 | not targeted |
