# Supplementary material for: Photocontrol of Axillary Bud Outgrowth by MicroRNAs: Current State-of-the-Art and Novel Perspectives Gained From the Rosebush Model
Source: Front Plant Sci. 2022 Jan 31;12:770363. doi: 10.3389/fpls.2021.770363 (PMC8841825; doi:10.3389/fpls.2021.770363)
Supplement: Supplementary file 2 [file Data_Sheet_1.docx]

**Supplemental Data 1: Sequences of mature miRNAs identified from small RNA sequencing and annotated from miRbase database (miRbase.org, Kozomara et al., 2019).**

>miR156z

ATTGGAGTGAAGGGAGCT

>miR172g-3p

GGAATCTTGATGATGCTGCAG

>miR172b

GGAATCTTGATGATGCTGCA

>miR166d-5p_2

GGAATGTTGTCTGGCTCGAGG

>miR166a

TCGGACCAGGCTTCATTCC

>miR172j

GCAGCATCATCAAGATTCCCA

>miR166e

GGACCAGGCTTCATTCCCC

>miR166e-3p

CTCGGACCAGGCTTCATTCCC

>miR172d_2

TGAGAATCTTGATGATGCTGCAT

>miR160h

TGCCTGGCTCCCTGTATGCCATT

>miR393b-5p

TCCAAAGGGATCGCATTGATCT

>miR159b-3p_1

ATTGGAGTGAAGGGAGCTCCA

>miR393a-3p_2

ATCATGCTATCCCTTTGGATT

>miR171l

CGAGCCGAACCAATATCACTC

>miR171c-5p_2

AGATATTGGTGCGGTTCAATC

>miR393-5p

TTCCAAAGGGATCGCATTGAT

>miR319c_1

TTGGACTGAAGGGAGCTCCT

>miR319_1

TTGGACTGAAGGGAGCTCC

>miR393a

TCCAAAGGGATCGCATTGATC

>miR393a-5p

TCCAAAGGGATCGCATTGATCC

>miR172a_2

AGAATCTTGATGATGCTGCA

>miR171b-3p

TTGAGCCGTGCCAATATCAC

>miR172i

AGAATCCTGATGATGCTGCAA

>miR393a_1

ATCCAAAGGGATCGCATTGATC

>miR393h

TTCCAAAGGGATCGCATTGATC

>miR393a_3

CATCCAAAGGGATCGCATTGA

>miR319b_1

CTTGGACTGAAGGGAGCTCCT

>miR171a-3p

TGATTGAGCCGCGCCAATATC

>miR166h-3p_1

TCTCGGACCAGGCTTCATTCC

>miR166e-5p

GGAATGTTGTCTGGCACGAGG

>miR160a-3p_2

GCGTATGAGGAGCCATGCATA

>miR160a-5p_1

GCCTGGCTCCCTGTATGCCAT

>miR172c-5p_3

AGCATCTTCAAGATTCACA

>miR828a_1

TCTTGCTCAAATGAGTATTCCA

>miR171a_3

TGATTGAGCCGTGCCAATAT

>miR169g

TAGCCAAGGATGACTTGCCTGC

>miR166g-5p_3

GAATGCTGTCTGGTTCGAGAC

>miR8175

GATCCCCGGCAACGGCGCCA

>miR166u

TCTCGGACCAGGCTTCATTC

>miR164b

TGGAGAAGCAGGGCACGTGC

>miR166

TCGGACCAGGCTTCATTCCCCC

>miR8051-5p

TAGTATGGTAGAAAGATTCA

>miR164f_1

TGGAGAAGCAGGGCACATGCT

>miR160g_2

TGCCTGGCTCCCTGGATGCCA

>miR408b_1

TGCACTGCCTCTTCCCTGGCT

>miR408d

TGCACTGCCTCTTCCCTGGC

>miR3711

TGGCGCTAGAAGGAGGGCCT

>miR530_2

TCTGCATTTGCACCTGCACCT

>miR156f

TTGACAGAAGAGAGAGAGCACA

>miR390a-5p

AAGCTCAGGAGGGATAGCGCC

>miR319f_1

ATTGGACTGAAGGGAGCTCC

>miR530a_2

TGCATTTGCACCTGCACCTC

>miR408-3p_2

ATGCACTGCCTCTTCCCTGGC

>miR396g-3p

GTTCAAGAAAGCTGTGGAAGA

>miR396g

TCCACAGGCTTTCTTGAACGG

>miR171b_3

TGATTGAGCCGCGTCAATATC

>miR827a

TTAGATGACCATCAACAAACA

>miR166h-5p

GGAATGTTGGCTGGCTCGAGG

>miR845

TGCTCTGATACCAATTGTTGG

>miR396h

TCCACAGCTTTCTTGAACTG

>miR396a-5p

TTCCACAGCTTTCTTGAACTG

>miR171_2

TTGAGCCGCGTCAATATCTCC

>miR171c_3

AGATTGAGCCGCGCCAATATC

>miR169v_1

CAGCCAAGGATGACTTGCC

>miR169b-5p

CAGCCAAGGATGACTTGCCGG

>miR171d_1

TTGAGCCGCGCCAATATCAC

>miR171f_3

TTGAGCCGCGCCAATATCACT

>miR171b-3p_3

TTGAGCCGTGCCAATATCACG

>miR396a-3p_4

GTTCAATAAAGCTGTGGGAA

>miR171e-5p

CGATGTTGGTGAGGTTCAATC

>miR3627d

TCCATCCTCCTGTGACATGAA

>miR6281

GTTAGAGATAGAGAGAGTGAG

>miR171e_1

TTATTGAACCGGACCAATATC

>miR319g

TTGGACTGAAGGGAGCTCCCA

>miR396a-3p_1

GTTCAATAAAGCTGTGGGAAG

>miR396a-3p_5

TTCAATAAAGCTGTGGGAAG

>miR169e_3

AGCCAAGGATGACTTGCCGG

>miR171f-5p_2

GGATATTGGTCCGGTTCAATA

>miR319p

TTTTGGACTGAAGGGAGCTCC

>miR396b-5p

TTCCACAGCTTTCTTGAACTT

>miR2109

TGCGAGTGTCTTCGCCTCTGA

>miR156c_1

TGACAGAAGAGAGGGAGCA

>miR156k_1

TGACAGAAGAGAGGGAGCAC

>miR169d-5p_1

TAGCCAAGGATGACTTGCCT

>miR390.1

TAAAGCTCAGGAGGGATAGCG

>miR164a_2

TGGAGAAGCAGGGCACTTGCT

>miR2111a-5p

TAATCTGCATCCTGAGGTTTA

>miR172a_3

AGAATCTTGATGATGCTGCAT

>miR172c-3p

AGAATCTTGATGATGCTGC

>miR171b

TGAGCCGTGCCAATATCACAT

>miR172e-3p

GGAATCTTGATGATGCTGCAT

>miR399e_5

TGCCAAAGGAGATTTGCTCGG

>miR398b_3

CGTGTTCTCAGGTCGCCCCTG

>miR160a-5p

TGCCTGGCTCCCTGTATGCCA

>miR160b_1

TGCCTGGCTCCCTGTATGCC

>miR160

TGGCATACAGGGAGCCAGGCA

>miR167d

TGAAGCTGCCAGCATGATCTGG

>miR167d-5p

TGAAGCTGCCAGCATGATCTG

>miR2275a-3p_2

TTTAGTTTCCTCCAATATCTTA

>miR397a_6

ATTGAGTGCAGCGTTGATGAA

>miR397-5p_1

ATTGAGTGCAGCGTTGATGA

>miR172c-5p

GTAGCATCATCAAGATTCACA

>miR172b-5p_2

GTAGCATCATCAAGATTCAC

>miR398b

TGTGTTCTCAGGTCGCCCCTG

>miR2275-3p

TTTGGTTTCCTCCAATATCTCG

>miR399f_3

TGCCAAAGGAGATTTGCCCGG

>miR399a_5

TGCCAAAGGAGAATTACCCTG

>miR2109-5p

TGCGAGTGTCTTCGCCTCTG

>miR319b-5p_2

GAGCTTTCTTCAGTCCACTC

>miR319a-3p

TTGGACTGAAGGGAGCTCCC

>miR319a

CTTGGACTGAAGGGAGCTCC

>miR319c_2

CTTGGACTGAAGGGAGCTCCC

>miR169u

CAGCCAAGGATGACTTGCCGT

>miR1030a

TCTGCATCTGCACCTGCACCA

>miR172e-3p_1

GAATCTTGATGATGCTGCAT

>miR390e

AGCTCAGGAGGGATAGCGCC

>miR156j_1

GTTGACAGAAGAGAGTGAGCAC

>miR169h_2

TAGCCAAGGATGACTTGCCTG

>miR399e

TGCCAAAGGAGATTTGCCCAG

>miR7125

CGAACTTATTGCAACTAGCTT

>miR172d-5p_5

GCGGCATCATCAAGATTCACA

>miR160b-3p

GCGTACGAGGAGCCAAGCATA

>miR172f

TGAATCTTGATGATGCCGCAC

>miR159c

TTTGGTTTGAAGGGGGCTCTG

>miR172a_1

GGGAATCTTGATGATGCTGCA

>miR397a_3

TCATTGAGTGCAGCGTTGATG

>miR399a_6

TGCCAAAGGAGAATTGCCCTG

>miR399_1

TGCCAAAGGAGAATTGCCC

>miR172a_4

AGAATCCTGATGATGCTGCAG

>miR395b-3p

AAGTGTTTGGGGGAACTC

>miR4995

AGGCAGTGGCTTGGTTAAGGG

>miR395l

CTGAAGTGTTTGGGGGAACCC

>miR398

TGTGTTCTCAGGTCACCCCT

>miR398a-3p_1

TGTGTTCTCAGGTCACCCCTT

>miR403-3p_1

TTAGATTCACGCACAAACTCG

>miR395d-5p_1

GTTCCCTTGACCACTTCATTG

>miR395a_5

CTGAAGTGTTTGGGGGAACTC

>miR169k_3

TGAGCCAAGGATGACTTGCCT

>miR395b_3

TGAAGTGTTTGGGGGAACTC

>miR395a_1

CTGAAGTGTTTGGGGGAACTCC

>miR156c

TTGACAGAAGAGAGAGAGCAC

>miR398b-3p_1

TTGTGTTCTCAGGTCACCCCT

>miR4414a-5p

AGCTGCTGACTCGTTGGTTCA

>miR167c_1

TGAAGCTGCCAGCATGATCTC

>miR169j-3p_1

GGCAGTCTCCTTGGCTATC

>miR395a-3p

CTGAAGTGTTTGGGGGGACCC

>miR156_2

TTGACAGAAGATAGAGAGC

>miR166a-3p

TCGGACCAGGCTTCATTCCCC

>miR5139

AAACCTGGCTCTGATACCA

>miR319a_1

TTGGACTGAAGGGAGCTCCCT

>miR168a-3p

CCCGCCTTGCATCAACTGAAT

>miR162a-5p_1

GGAGGCAGCGGTTCATCGATC

>miR162a-5p

TGGAGGCAGCGGTTCATCGATC

>miR399b

TGCCAAAGGAGAGTTGCCCTG

>miR5138

AAAAATCGTTAGGCGCTA

>miR157d-3p

GCTCTCTATGCTTCTGTCATC

>miR6300

GTCGTTGTAGTATAGTGG

>miR171f-3p

TTGAGCCGTGCCAATATCACA

>miR169f.1

TAGCCAAGGATGACTTGCCTA

>miR168-3p

TCCCGCCTTGCATCAACTGAAT

>miR162a-3p

TCGATAAACCTCTGCATCCAG

>miR162-3p

TCGATAAACCTCTGCATCCA

>miR166m_2

CGGACCAGGCTTCATTCCCC

>miR845b-3p

TCGCTCTGATACCAAATGATG

>miR168a-5p

TCGCTTGGTGCAGGTCGGGAA

>miR168

TCGCTTGGTGCAGGTCGGGA

>miR168-5p

TCGCTTGGTGCAGGTCGGGAAC

>miR157a-5p

TTGACAGAAGATAGAGAGCAC

>miR157d

TGACAGAAGATAGAGAGCAC

>miR171a-3p_1

TGATTGAGCCGCGCCAATAT

>miR156g_1

ACAGAAGATAGAGAGCACAG

>miR156d-3p_4

GCTCTCTATGCTTCTGTCATCA

>miR159a_1

TTTGGATTGAAGGGAGCTCTA

>miR160h_1

TGCCTGGCTCCCTGCATGCCA

>miR477e

CTCTCCCTCAAGGGCTTCTA

>miR394a_1

TTGGCATTCTGTCCACCTCC

>miR167a

TGAAGCTGCCAGCATGATCTAA

>miR167d_1

TGAAGCTGCCAGCATGATCT

>miR171b_2

TGATTGAGCCGTGCCAATATC

>miR166h-3p

TCGGACCAGGCTTCATTCCC

>miR8626

GTCAGCAGCCGCCGCCGC

>miR164a_1

CACGTGCTCCCCTTCTCCAAC

>miR166-3p

ATTTCGGACCAGGCTTCATTCCCC

>miR167a-5p

TGAAGCTGCCAGCATGATCTA

>miR156f-3p_4

GCTCACTCTCTATCTGTCACC

>miR167b-3p_3

GGTCATGCTCTGACAGCCTCACT

>miR171c-5p

TATTGGTGCGGTTCAATCAGA

>miR156b-3p

TGCTCACTCTCTATCTGTCACC

>miR1510b-3p

TGTTGTTTTACCTATTCCACC

>miR396b

TTCCACAGCTTTCTTGAACT

>miR396e

TTCCACAGCTTTCTTGAACTGT

>miR156a-5p

TGACAGAAGAGAGTGAGCAC

>miR156a

TGACAGAAGAGAGTGAGCACA

>miR164a_4

TGGAGAAGCAGGGCACGTGCA

>miR164_1

TGGAGAAGGGGAGCACGTGCA

>miR3623-5p

TCACAAGTTCATCCAAGCACCA

>miR164e-5p

TGGAGAAGCAGGGCACGTGCAA

>miR156k_2

TTGACAGAAGAGAGTGAGCAC
