# Supplementary material for: Photocontrol of Axillary Bud Outgrowth by MicroRNAs: Current State-of-the-Art and Novel Perspectives Gained From the Rosebush Model
Source: Front Plant Sci. 2022 Jan 31;12:770363. doi: 10.3389/fpls.2021.770363 (PMC8841825; doi:10.3389/fpls.2021.770363)
Supplement: Supplementary file 3 [file Data_Sheet_2.docx]

Supplemental Method I

**Plant material and culture**

*Rosa* ‘Radrazz’ is a triploid rosebush, known to be disease resistant, with a primary axis made up of 8 to 12 phytomers, ending in a flower bud. Each phytomer has a bud located in the axil of a leaf, itself made up of 3 to 7 leaflets. Mother-plants of *Rosa* ‘Radrazz’, all being of the same genotype, are used to provide single-node cuttings.

Single-node cuttings are taken from the median part of the axes carrying a leaf with 5 to 7 leaflets and a dormant bud at its axil. Cuttings are then planted in FERTISS clods (FERTISS, SAS, Boulogne Billancourt, France) and placed under "rooting" tunnels with high hygrometry level (80-90%) for 3 to 5 weeks. Once well rooted, the cuttings are repotted in 500mL pots containing a mixture of peat, coconut fiber and perlite (70/20/10, V / V / V), then are placed under greenhouse conditions (temperature 25 ± 5 ° C, light intensity ~ 250 ± 50 µmol.m-2.s-1, long day photoperiod (16h day / 8h night) and sub-irrigated with a commercial nutrient solution (Peter Excel - Scott). When the bud of the cutting has grown out and has produced a new axis of a few centimeters long (about a week after repotting), the leaf of the cutting is gently removed to limit the development of pathogens. Likewise, phytosanitary treatments are carried out regularly to limit the appearance of aphids, thrips, mites or pathogens such as powdery mildew.

When cuttings have produced an axis bearing 4 leaves with 5 visible leaflets after 3 or 4 weeks of culture, the plants are transferred to a growth chamber where the growing conditions are under control. In these chambers, the temperature is fixed at 22 °C, the hygrometry at 70%, the plants are placed under LED ramps of white light with a PPFD of approximately 250 µmol.m^-2^.s^-1^. The plants are left 3 to 4 days for acclimation and until they have produced a flower bud at the extremity of their axis. When the flower bud is just visible (VFB stage, Girault et al., 2008), plants are beheaded, in order to lift apical dominance and to synchronize the outgrowth of distal buds in all plants.

***In planta* experiments**

Plants at VFB stage were beheaded by cutting the upper part of the plant axis at 1 cm above the fourth basal leaf-bearing five-leaflets using a razor blade. Upon beheading (t0) and six hours (t6) after beheading, axillary buds located at the third basal leaf-bearing five-leaflets were harvested, frozen in liquid nitrogen and stored at -80°C. These time-points were chosen according to previous works on bud outgrowth light-control (Girault et al., 2010; Barbier et al., 2015; Roman et al., 2016), showing rapid transcriptional control by light conditions after beheading. Three biological replicates were made for each condition using 25 plants.

It has been shown by Girault et al. (2008), that axillary bud outgrowth in rosebush occurs when plants are exposed to light, whereas it is fully inhibited by darkness. This axillary bud outgrowth is characterized by the elongation of the buds and the resumption of organogenesis activity. In order to validate our experimental conditions, its elongation and its organogenesis were recorded over time (until 96h after beheading).

We have observed an increase in the number of leaf-like organs in third bud for plants under light condition, from 8 preformed leaves counted just after decapitation (t0) to 13 leaf-like organon average at 96 hours after decapitation (t96), the measurements at 72h and 96h being significantly different compared to t0 (data not shown).

The length of the third bud of plants placed under light was evaluated over 4 days after decapitation. We can observe an increase in the average bud length for the light-treated plants, going from 1.5 mm (t0) to 3.5 mm (t96) with the 48h, 72h and 96h points significantly different compared to the t0 point (data not shown).

We have validated that we reproduce a growth dynamic (increase in organogenesis and elongation) in the third bud of plants placed in the light identical to that described by Girault et al., (2008).

**Total RNA extraction, small RNA sequencing and annotation**

About 20mg of frozen buds were ground in liquid nitrogen and used for total RNA isolation using a method developed by Barbier et al., (2019). Quantity and quality of RNA were evaluated using Nanodrop One (Thermo Scientific), agarose gel and Agilent bio-analyser for RIN measurement. 1µg of total RNA was sent to BGI Genomics (Beijing Genomics Institute, China) for small RNA library construction and deep sequencing using Illumina technology on BGISEQ-500. Annotation of miRNAs was performed using annotated *Arabidopsis thaliana* miRNAs on miRbase database (miRbase.org, Kozomara et al., 2019)

**Target prediction**

“A Plant Small RNA Target Analysis Server (2017 Update)” psRNATarget was used to identify targets of annotated *Rosa* miRNAs (Dai X. et al., 2018a). Default parameters were used with changes of maximum expectation at 3.5 to ensure more rigorous predictions.
